# Case Report: Identification of a Novel *ODAD3* Variant in a Patient With Primary Ciliary Dyskinesia

**DOI:** 10.3389/fgene.2021.652381

**Published:** 2021-02-26

**Authors:** Rongchun Wang, Danhui Yang, Ting Guo, Cheng Lei, Xu Chen, Xi Kang, Jie Qing, Hong Luo

**Affiliations:** ^1^Department of Pulmonary and Critical Care Medicine, The Second Xiangya Hospital, Central South University, Changsha, China; ^2^Research Unit of Respiratory Disease, Central South University, Changsha, China; ^3^Hunan Diagnosis and Treatment Center of Respiratory Disease, Changsha, China

**Keywords:** primary ciliary dyskinesia, *ODAD3*, *CCDC151*, sinusitis, bronchiectasis, dextrocardia, infertility

## Abstract

**Background:** *ODAD3* encodes a protein of 595 amino acids and contain three highly conserved coiled-coil domains, which is essential for cilia axoneme dynein arm assembly and docking. Primary ciliary dyskinesia (PCD) of *ODAD3* deficiency are rarely reported. Female infertility in PCD related to *ODAD3* variants has not been reported.

**Methods:** Whole-exome and Sanger sequencing were used to identify the disease-related gene of the patient with PCD in a consanguineous Chinese family. Domain analysis was applied to predict the impact of the variant on ODAD3 protein.

**Results:** The 35 year-old female patient exhibited chronic sinusitis, diffuse bronchiectasis, dextrocardia and infertility. We identified a novel homozygous variant in *ODAD3*, c.1166_1169dupAGAC, p.(Leu391Aspfs^*^105) in the PCD patient by exome sequencing and Sanger sequencing. This frameshift variant was predicted to be disease causing by bioinformatics analysis and was also not presented in the current authorized large genetic databases.

**Conclusions:** Our study enriches the genetic spectrum and clinical phenotypes of *ODAD3* variants in PCD and provide more evidence for future genetic counseling and gene-targeted therapy for this disease.

## Introduction

Primary ciliary dyskinesia (PCD, MIM 244400) is a clinically and genetically heterogeneous motile ciliopathy characterized by recurrent upper and lower respiratory infections, subfertility and laterality defect (Mirra et al., 2017). The estimated prevalence of PCD is 1:10 000 to 1:20 000 live-born children (Afzelius and Stenram, 2006). So far, more than 47 genes associated with cilia structure or function dysfunction have been identified causative for PCD (Wallmeier et al., 2020).

*ODAD3* (Outer dynein arm docking complex subunit 3; formerly known as *CCDC151*; coiled-coil domain containing 151) encodes a protein of 595 amino acids which contains three highly conserved coiled-coil domains. This protein was initially recognized as a potential ciliome gene due to its expression in human ciliary axonemes (Ostrowski et al., 2002) and it is essential for cilia axoneme dynein arm assembly and docking (Hjeij et al., 2014). Up to now, PCD caused by *ODAD3* variants (MIM 616037, CILD30) are rarely reported and female infertility has not been identified in PCD caused by *ODAD3* variants.

In the present study, a novel variant in *ODAD3* was identified in a female PCD patient with infertility from a Chinese consanguineous family.

## Case Presentation

**Figure 1:**
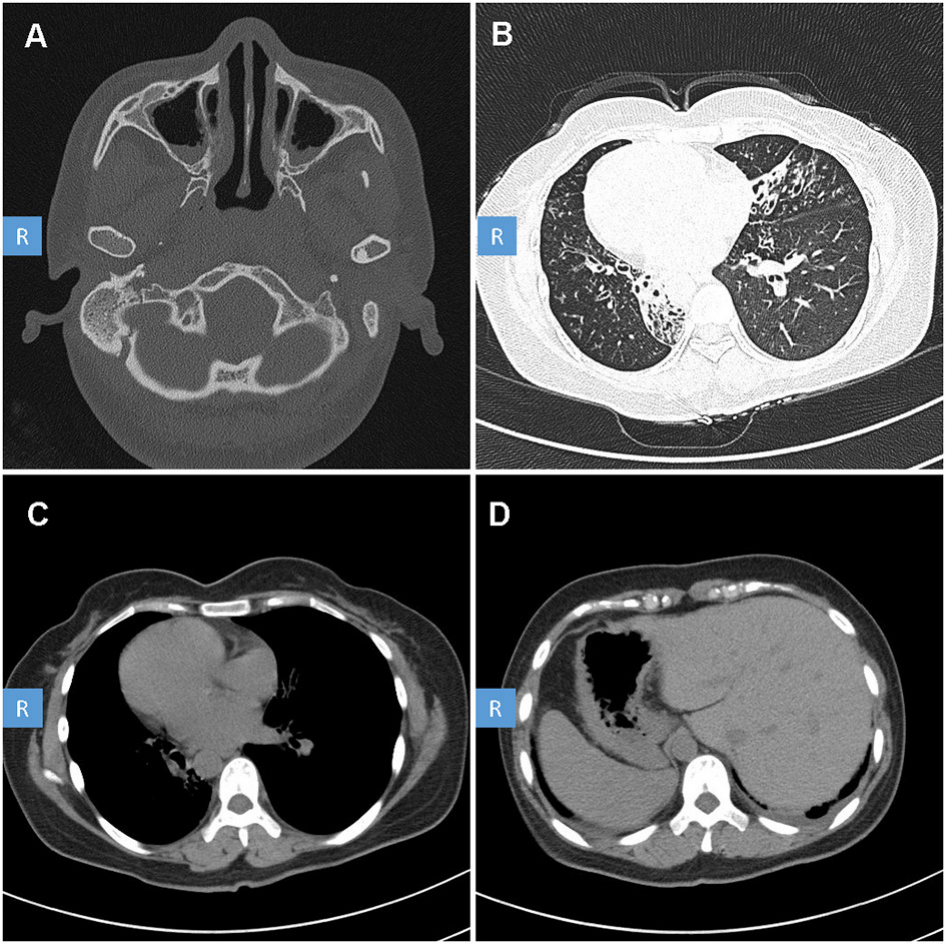
High-resolution computed tomography (HRCT) scan of the patient with PCD. CT scan showed: **(A)** Sinusitis. **(B)** Diffuse bronchiectasis. **(C)** Right-side heart. **(D)** Abdominal organ heterotaxy: liver in the left side, stomach and spleen in the right side.

The patient is a 35 year-old female. She was admitted to our hospital because of chronic cough, purulent sputum, nasal congestion for over 30 years and dyspnea for about 5 years. She has been inflicted by recurrent infections of the lower and upper airways since newborn. And he has been married for 10 years but without pregnancy and was diagnosed with infertility. She had no history of drowning, measles, polio, tuberculosis, immunodeficiency, connective tissue disease, etc. Her family members denied any obvious respiratory or other disease history. Physical examination indicated right-side heart sound. High resolution computed tomography of this patient revealed chronic sinusitis (Figure 1A), diffuse bronchiectasis (Figure 1B), situs inversus: heart dextroversion (Figure 1C) and abdominal organs in the reverse location (Figure 1D). Lung function test revealed a moderate to severe mixed (restrictive and obstructive) ventilation dysfunction. Her nasal nitric oxide concentration (nNO) (6 nl/min) was far below the reference nNO cutoff value of PCD (77 nl/min) (Leigh et al., 2013).

## Methods

### Ethical Compliance

This study was approved by the Review Board of the Second Xiangya Hospital of Central South University in China. Written informed consent was obtained from the patient.

### DNA Extraction and Variant Analysis

We extracted genomic DNA from peripheral blood of the proband and healthy control, using a QIAamp DNA Blood Mini Kit (250) (QIAGEN, Valencia, CA). Then we captured the DNA of the proband with the Agilent SureSelect Human All Exon V5 Kit (Agilent, California, USA) and sequenced on Illumina Hiseq 4000 (Illumina Inc., San Diego, USA).

The valid sequencing reads were aligned to the NCBI human reference genome (GRCh37/hg19) by the Burrows Wheeler Aligner software (Li and Durbin, 2010). ANNOVAR is used to do annotation for Variant Call Format file (Wang et al., 2010). We classified the variants as pathogenic, likely pathogenic and uncertain significance, likely benign, benign according to American College of Medical Genomics (ACMG) guidance (Richards et al., 2015).

**Figure 2:**
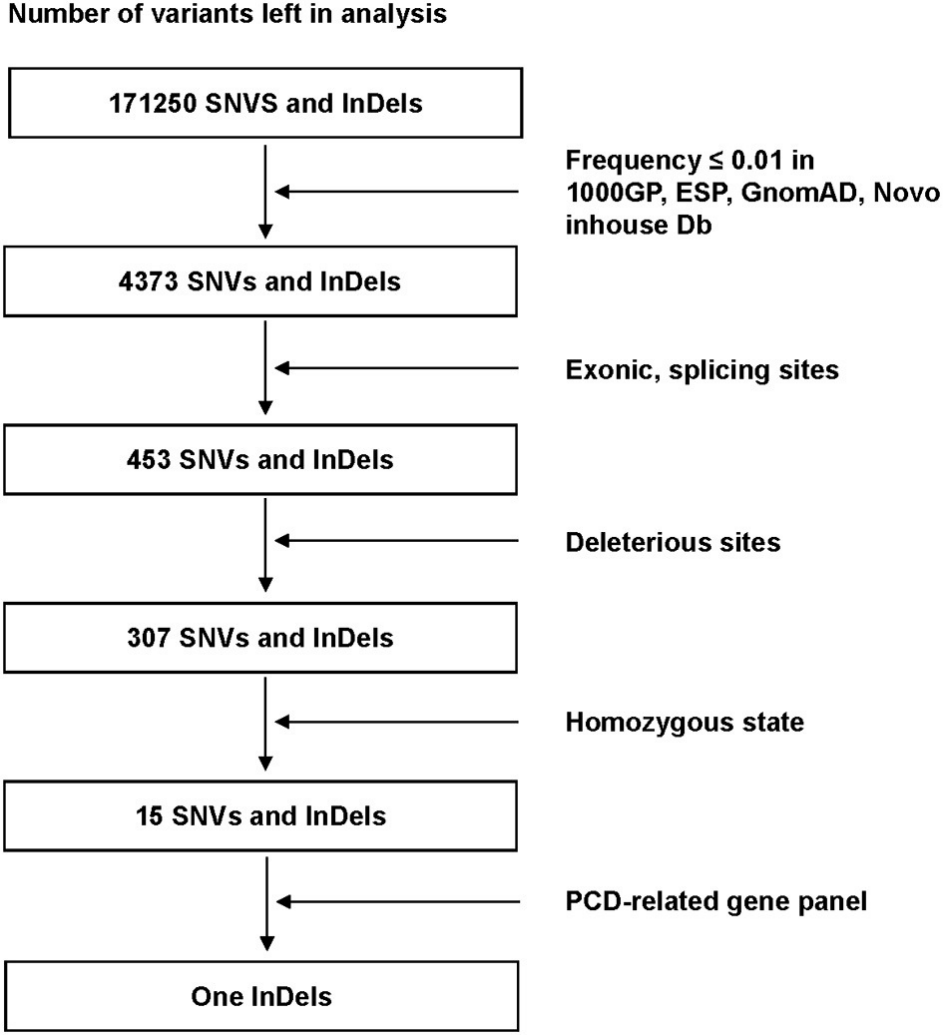
Filter process performed to all variants in the alignment SNVs and InDels. Only one InDels (*ODAD3*, NM_145045.4: c.1166_1169dup) left after all filter steps. SNVs, single-nucleotide variants: InDels, insertions and deletions; 1000GP, 1000 Genomes Project; ESP, Exome Sequencing Project; GnomAD, Genome Aggregation Database; PCD, Primary ciliary dyskinesia.

We filtered single-nucleotide variants (SNVs) and short insertions and deletions (InDels) as follows (Figure 2): (1) High-frequency (minor allele frequency > 0.01) variants found in 1000 Genomes Project, Exome Sequencing Project (ESP), Genome Aggregation Database (gnomAD) and Novo inhouse Database, were excluded. (2) Variants within Exonic, splicing sites were included from subsequent analysis. (3) Bioinformatics programs: Mutation Taster, Polyphen2, SIFT, CADD, and LoFtool were used to predict the possible impacts of SNVs. (4) Homozygous variants were retained to be filtered by a PCD-related gene panel including 47 identified genes (Supplementary Table 1). Sanger sequencing was performed in the patient to validate the mutation. The location of the mutation was analyzed by SMART program (http://smart.embl-heidelberg.de). The DNA samples from the patient's parents were not available. The primer sequences were designed as follows: forward, 5′-ATTCTAAGACCGCTGTGTCCC-3′; reverse, 5′ TTGCACAGCAATGTATGGGG-3′.

## Results

For the DNA sample of the patient, exome sequencing generated an average of 14.5 Gb data with an ~99% coverage and a depth of >50×. After alignment and frequency filter, 4,373 non-synonymous variants (SNVs and InDels) were further analyzed and 453 variants in exons or in canonical splice sites (splicing junction 10 bp) were further analyzed. Fifteen SNVs and InDels in homozygous state were left and then filtered by the PCD-related gene panel. Finally, only one homozygous variant, *ODAD3*, NC_000019.9:g.11533477_11533480dup, NM_145045.4: c.1166_1169dupAGAC, NP_659482.3: p.(Leu391Aspfs^*^105), which caused frameshift insertion, passed the filtration.

**Figure 3:**
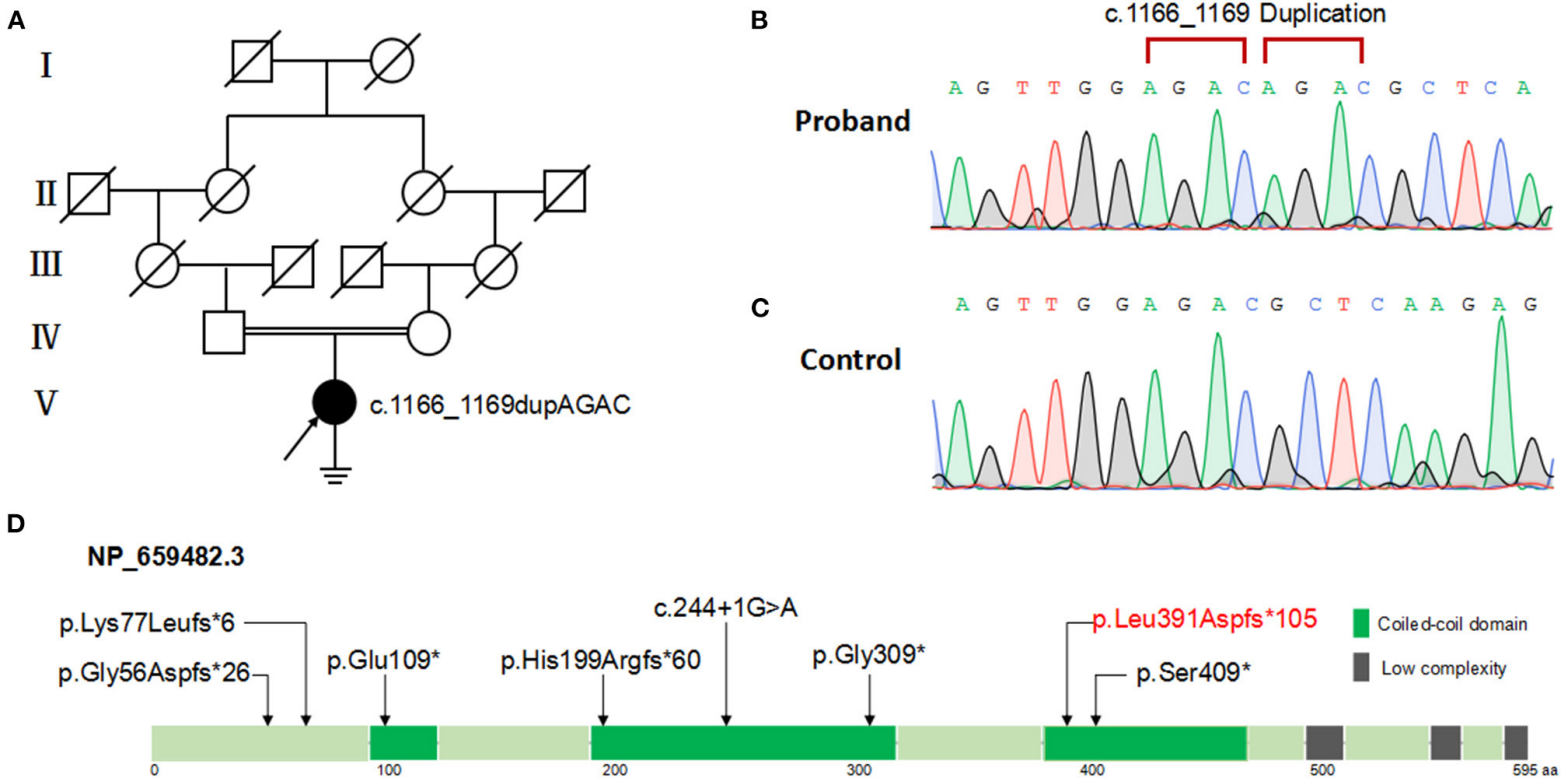
Pedigree and *ODAD3* variants analysis of the patient with PCD. **(A)** Family pedigree of the patient with PCD. Filled symbol represents the affected member, empty symbol represents unaffected members, slashed symbols represent deceased members. Squares, males; circles, females. The patient (V:1) is indicated by a black arrow. **(B)** Patient with *ODAD3* c.1166_1169dupAGAC alleles. **(C)** Normal control with wild-type alleles. **(D)** The location of the mutations is shown within the third coiled-coil domain of the 595-amino-acid ODAD3 protein (Refseq NP_659482.3) model. Green boxes indicate coiled-coil domain; gray boxes indicate low-complexity repeat regions.

This variant was in accord with the hereditary mode from consanguineous family (Figure 3A) and was validated via Sanger sequencing (Figures 3B,C). According to ACMG guidelines (Richards et al., 2015), this variant was classified into pathogenic (meeting criteria of PVS1, PM2, PM3). The location of the variant is within the third coiled-coil domain of the 595-amino-acid ODAD3 protein (Refseq NP_659482.3) analyzed by SMART program (http://smart.embl-heidelberg.de) (Figure 3D).

## Discussion and Conclusion

This study describes a female with PCD that carried a novel homozygous variant in the *ODAD3* gene. This patient in our study exhibited chronic sinusitis, bronchiectasis, situs inversus, and infertility. Our case enriches the mutation spectrum and clinical phenotypes of *ODAD3* variants in PCD.

PCD is a motile ciliopathy involving multiple organs or system, especially in respiratory tract. Cilia can be classified into two types according their motility: motile cilia with 9+2 or 9+0 microtube structure and immotile cilia with 9+0 microtube (Satir and Christensen, 2007). Motile cilia has highly conserved cell structure among different species and extensively exists on airway epithelium, germinal center in fetal period, oviduct and sperm flagella (Bayless et al., 2019). The critical pathogenesis of PCD lies in the abnormality of motile cilia structure or function or ciliogenesis, which accounts for ineffective cilia clearance, abnormal left–right asymmetry and subfertility (Bustamante-Marin and Ostrowski, 2017).

To date, a total of seven likely loss-of-function variants in *ODAD3* have been reported in PCD (Supplementary Table 2). The reported *ODAD3* variants including three non-sense variants: p.(Gly309 ^*^) (Hjeij et al., 2014); p.(Ser409^*^) (Hjeij et al., 2014); p.(Glu109^*^) (Zhang et al., 2019); three frame-shifting variants: p.(Gly56Aspfs^*^26) (Deng et al., 2020); p.(Lys77Leufs^*^6) (Mani et al., 2020); p.(His199Argfs^*^60) (Olm et al., 2019) and one splice site variant: c.244+1G>A (Monies et al., 2019). The only two hitherto reported female patients (10 week and 1 year-old, respectively) did not reach reproductive age. infertility has not been determined in the previously reported patients. However, it means that it was not reported (and probably not even investigated in adult patients) and not that it was not present. Therefore, assessing and reporting infertility in patients affected by PCD is essential, whenever possible. In our study, we detected a novel homozygous variant of *ODAD3*, p.(Leu391Aspfs^*^105) which was likely to be PCD disease-causing. The patient in our study exhibited typical PCD phenotypes including chronic sinusitis, bronchiectasis, situs inversus and infertility. The frame-shift variant results in an incorrect and premature termination of translation of ODAD3 protein which may undergo non-sense-mediated decay, indicating a loss of function of *ODAD3* in outer dynein arm (ODA) assembly and docking.

Infertility is more common in males than females in PCD (Wallmeier et al., 2020). The prevalence of infertility among PCD females is unclear, partly due to insufficient awareness of clinicians. A cohort study (Vanaken et al., 2017) among PCD reported 61% (22/36) women are considered infertile related with 13 genes such as *CCDC39, CCDC40, DNAH5*, which affect motile cilia structure. *CCDC39* and *CCDC40*, similar to *ODAD3*, also contain coiled-coil domains and their defects display disorganization of microtubule doublets and central pair in cilia (Becker-Heck et al., 2011; Merveille et al., 2011). *DNAH5* is the ODA component of motile cilia and its defect lead to ciliary dyskinesia (Hornef et al., 2006). These data indicate that those genes impacting motile cilia structure, may related to female infertility. Our study is the first to report infertility in female PCD patient with *ODAD3* deficiency. The frameshift *ODAD3* variant may lead to ODA defects and then impede the oocytes transportation across the oviduct, which finally results in infertility. As there are currently no way for women to test their potentially PCD-related fertility before trying to conceive, it is necessary for clinicians to pay more attention to fertility status of PCD. Fertility counseling and appropriate assisted reproductive technologies should be included in the standard PCD patient care.

Animal models of deficient *ODAD3* demonstrated phenotypes of ciliary dyskinesia and laterality defects similar to human. *Odad3* in vertebrates is homologous to the *Chlamydomonas ODA10* gene which is also associated with cilia outer ODA assembly (Dean and Mitchell, 2013). *ODAD3* is involved in regulating intraflagellar transport (IFT)-dependent dynein arm assembly according to previous research in *drosophila*, zebrafish, and mice (Alsaadi et al., 2014; Hjeij et al., 2014; Jerber et al., 2014). *ODAD3*-knockdown zebrafish had motile ciliary dysfunction in the pronephros and Kupffer's vesicle, and showed abnormal left–right asymmetry (Hjeij et al., 2014). In mice, *Odad3* is expressed in embryonic nodes and ependymal cells, and deficient *Odad3* may result in ciliary dyskinesia and laterality defects (Jerber et al., 2014). Whether *ODAD3* defect leads to infertility in animal remains unknown and need further research to recapture phenotype in human.

In conclusion, combined clinical profile and sequencing data, we suggest that novel homozygous variant of *ODAD3*, p.(Leu391Aspfs^*^105), is one of the pathogenic cause of PCD. This is also the first report about female infertility in PCD of *ODAD3* deficiency. Our study enriches the genetic spectrum and clinical phenotypes of *ODAD3* variants in PCD and provides more evidence for future genetic counseling and gene-targeted therapy for this disease.

## Data Availability Statement

The datasets for this article are not publicly available due to concerns regarding participant/patient anonymity. Requests to access the datasets should be directed to the corresponding author.

## Ethics Statement

The studies involving human participants were reviewed and approved by Review Board of the Second Xiangya Hospital of Central South University in China. The patients/participants provided their written informed consent to participate in this study. Written informed consent was obtained from the individual(s) for the publication of any potentially identifiable images or data included in this article.

## Author Contributions

HL and JQ designed the study. RW, DY, TG, and CL performed the genetic analysis and bioinformatics evaluations. RW and DY drafted the manuscript. HL, JQ, XC, and XK conducted the clinical evaluations. All authors analyzed the data and approved the final manuscript.

## Conflict of Interest

The authors declare that the research was conducted in the absence of any commercial or financial relationships that could be construed as a potential conflict of interest.
